# Synchronicity in Post‐Jungian Astrology: A Cosmological Quest

**DOI:** 10.1111/1468-5922.70016

**Published:** 2025-09-13

**Authors:** Jingchao Zeng, Nathan Fraikin

**Affiliations:** ^1^ China; ^2^ France; ^3^ UK

**Keywords:** astrology, Carl Jung, cosmology, divination, post‐Jungian astrology, synchronicity, astrologie, synchronicité, Jung, astrologie post‐jungienne, cosmologie, divination, Astrologie, Synchronizität, Jung, post‐jungianische Astrologie, Kosmologie, Wahrsagerei, astrologia, sincronicità Jung, astrologia post‐junghiana, cosmologia, divinazione, астрология, синхронистичность, Юнг, пост‐юнгианская астрология, космология, прорицание, astrología, sincronicidad, Jung, astrología post‐Junguiana, cosmología, adivinación, 星象学, 共时性, 荣格, 后荣格星象学, 宇宙论, 占卜

## Abstract

Jung promoted the idea of synchronicity in 1928, in the context of discussing the Chinese way of thinking. From 1928 to 1951, Jung’s informal formulations led to more than one understanding of synchronicity. He even tried to apply synchronicity to an astrological experiment. Yet, the experimental results reveal that synchronicity cannot be verified by natal astrology. However, post‐Jungian astrologers still pursue synchronicity in their astrological theories. In this article, Jung’s synchronicity theory is reviewed again to reassert the threshold of Jung’s original conceptualization of synchronicity as a form of empirical phenomena. From there, the article focuses on the reception of the concept among two post‐Jungian authors: Liz Greene and Richard Tarnas. They overlooked, each in their own way, Jung’s cautious epistemological attitude towards synchronicity in order to establish a grander cosmology for astrology. Finally, we suggest an alternative approach to discovering authentic synchronicity in astrology beyond the cosmological/metaphysical approach—by emphasizing the numinous foundation of the unconscious.

## Abstract (fr)

Jung a promu l'idée de synchronicité en 1928, dans le contexte d’une discussion sur la pensée chinoise. De 1928 à 1951, les différentes formulations informelles sur ce thème de Jung ont conduit à plusieurs façons de comprendre la synchronicité. Il a même essayé d'appliquer la synchronicité à une expérience astrologique. Pourtant, les résultats expérimentaux révèlent que la synchronicité ne peut pas être vérifiée par l'astrologie natale. Cependant, les astrologues post‐jungiens poursuivent encore la synchronicité dans leurs théories astrologiques. Dans cet article, la théorie de la synchronicité de Jung est réexaminée pour réaffirmer le seuil de la conception originale de la synchronicité par Jung comme une forme de phénomène empirique. À partir de là, l'article se concentre sur la réception du concept chez deux auteurs post‐jungiens: Liz Greene et Richard Tarnas. Ils ont négligé, chacun à sa manière, l'attitude épistémologique prudente de Jung envers la synchronicité afin d'établir une cosmologie plus grandiose pour l'astrologie. Enfin, nous suggérons une approche alternative pour découvrir une synchronicité authentique en astrologie au‐delà de l'approche cosmologique/métaphysique—en mettant l'accent sur le fondement numineux de l'inconscient.

## Abstract (de)

Jung propagierte die Idee der Synchronizität 1928 im Kontext einer Diskussion über die chinesische Denkweise. Von 1928 bis 1951 führten Jungs informelle Formulierungen zu mehr als einem Verständnis von Synchronizität. Er versuchte sogar, Synchronizität auf ein astrologisches Experiment anzuwenden. Die experimentellen Ergebnisse zeigen jedoch, daß Synchronizität nicht durch Geburtsastrologie verifiziert werden kann. Post‐jungianische Astrologen verfolgen jedoch weiterhin die Synchronizität in ihren astrologischen Theorien. Dieser Artikel untersucht Jungs Synchronizitätstheorie erneut, um die Grenzen von Jungs ursprünglicher Konzeptualisierung von Synchronizität als empirisches Phänomen zu bekräftigen. Anschließend konzentriert sich der Artikel auf die Rezeption des Konzepts bei zwei post‐jungianischen Autoren: Liz Greene und Richard Tarnas. Sie übersahen, jeder auf seine Weise, Jungs vorsichtige erkenntnistheoretische Haltung gegenüber Synchronizität, um eine umfassendere Kosmologie für die Astrologie zu etablieren. Abschließend schlagen wir einen alternativen Ansatz zur Entdeckung authentischer Synchronizität in der Astrologie vor, der über den kosmologischen/metaphysischen Ansatz hinausgeht – indem wir die numinosen Grundlagen des Unbewußten betonen.

## Abstract (it)

Jung ha presentato l'idea della sincronicità 1928, all'interno di una riflessione sul pensiero cinese. In seguito, ha cercato di applicare la sincronicità a un esperimento astrologico. Però i risultati dell'esperimento dimostrarono che la sincronicità non può essere verificata con l'astrologia natale. Ad ogni modo, gli astrologi post‐junghiani ancora perseguono il tema della sincronicità nelle loro teorie astrologiche. In questo articolo, viene nuovamente considerata la teoria della sincronicità di Jung per riaffermare il principio di questa concettualizzazione junghiana che vede la sincronicità come una possibile forma di manifestazione dei fenomeni empirici. Da qui, l'articolo analizza come questo concetto è stato recepito da due autori post‐junghiani: Liz Greene e Richard Tarnas. Loro hanno entrambi tralasciato l'attitudine epistemologica di Jung, il suo rigoroso atteggiamento verso la sincronicità, per creare una sproporzionata cosmologia astrologica. Per concludere, suggeriamo un approccio alternativo alla scoperta della autentica sincronicità nell'astrologia, che vada oltre la visione cosmologica/metafisica ed enfatizzi il fondamento numinoso dell'inconscio.

## Abstract (ru)

Юнг выдвинул идею синхронистичности в 1928 году в контексте обсуждения китайского образа мышления. Попытки Юнга дать определение понятию синхронистичности, предпринимаемые им в период с 1928 по 1951 год, привели к неоднозначности в понимании этого термина. Он даже попытался применить принцип синхронистичности к астрологическому эксперименту. Однако результаты эксперимента продемонстрировали, что синхронистичность не получается подтвердить натальной астрологией. Тем не менее, пост‐юнгианские астрологи продолжают говорить о синхронистичности в своих астрологических теориях. Авторы данной статьи приводят обзор Юнговой теории синхронистичности с целью напомнить об исходном понимании Юнгом этого феномена как разновидности эмпирического явления. Затем фокус статьи смещается на восприятие этой концепции двумя пост‐юнгианскими авторами, Лиз Грин и Ричардом Тарнасом. Каждый из них по‐своему пренебрегает свойственной Юнгу осторожностью в отношении эпистемологии синхронистичности и предлагает более масштабную космологию для астрологии. Наконец, мы предлагаем альтернативный подход к поиску подлинной синхронистичности в астрологии, выходящий за рамки космологического/метафизического понимания и опирающийся на нуминозные основания бессознательного.

## Abstract (es)

Jung promovió la idea de sincronicidad en 1928, en el contexto de la discusión sobre la forma de pensar en China. De 1928 a 1951, las formulaciones informales de Jung dieron lugar a más de una comprensión acerca de la sincronicidad. Incluso intentó aplicar la sincronicidad a un experimento astrológico. No obstante, los resultados experimentales revelan que la sincronicidad no puede ser verificada por la astrología natal. Sin embargo, los astrólogos post‐Jungianos continúan aplicando la sincronicidad en sus teorías astrológicas. En este artículo, la teoría de la sincronicidad de Jung es nuevamente revisada con el objetivo de reafirmar el umbral de la conceptualización original de Jung de la sincronicidad como una forma de fenómeno empírico. A partir de esto, el artículo se centra en cómo dicho concepto fue acuñado por dos autores post‐Jungianos: Liz Greene y Richard Tarnas. Ambos pasaron por alto, cada uno a su manera, la cautelosa actitud epistemológica de Jung hacia la sincronicidad con el fin de establecer una cosmología más amplia para la astrología. Por último, sugerimos un enfoque alternativo para descubrir la auténtica sincronicidad en la astrología, más allá del enfoque cosmológico/metafísico, enfatizando el fundamento numinoso del inconsciente.

## Abstract (zh)

后荣格星象学中的共时性:一场宇宙论的追寻

荣格于1928年在探讨中国思维方式时提出“共时性”概念。从1928年至1951年, 他的非正式表述带出关于共时性的多种理解。他甚至尝试将共时性应用于一项占星实验。然而, 实验结果显示, 出生占星术无法验证共时性。尽管如此, 后荣格星象学家仍在各自理论中追索共时性。本文重新检视荣格的共时性理论, 重申其原始界定, 即共时性应被视为经验性的现象。随后, 文章聚焦于两位后荣格学者:Liz Greene 与 Richard Tarnas, 对这一概念的接受方式。二人各自, 以自己的方式, 忽略了荣格在对待共时性时, 在认识论上的谨慎, 以构筑更为宏大的星象宇宙论。最终, 我们提出了另一种方法, 以在星象学中发现真实共时性, 这种方法通过强调无意识的神圣根基, 来超越宇宙论的/形而上的方法。

## Jung’s Synchronicity as a Concept of Empirical Phenomena

In 1952, Jung published *Synchronicity: An Acausal Connecting Principle* (Jung, 1952/1975). Throughout his essay, Jung strives to explain meaningful coincidences which cannot be reduced to causality. Thus, he formulates his concept of synchronicity as: “the simultaneous occurrence of a certain psychic state with one or more external events which appear as meaningful parallels to the momentary subjective state” (Jung, 1952/1975, para. 850).

Jung (1952/1975) tries to draw upon statistics looking for a proof of such acausal connections (para. 827). He attributes to Joseph Banks Rhine the first decisive evidence of synchronistic events. Jung considers that across Rhine’s experiments, some significant statistical connections between the subject and the object studied can transcend the limit of time and space (para. 840).

Jung (1952/1975) even set up his own “astrological experiment” as an attempt to apply statistics to include the intuitive methods of “grasping the whole situation” like astrology into science. In pursuit of the unification of the intuitive paranormal and science (para. 864), he studied statistically the correlation between the horoscopes of married couples and traditional astrological aspects of marriage (Jung, 1952/1975, paras. 869, 878; Zeng, 2024, p. 189). However, statistically insignificant results revealed that such connection between married couples and astrological aspects is merely chance (Jung, 1952/1975, para. 901).

Jung eventually realized that the quantitative approach of statistics is unable to establish scientifically the principle of synchronicity (Jung, 1952/1975, para. 877). Consequently, he conducted a second, but this time qualitative experiment. Here, Jung had participants randomly select couple horoscopes (drawing 10 out of 200) and examined the primary astrological aspects therein to identify the connection between the major aspects with the participants’ psychological states (Jung, 1952/1975, paras. 896–899). Under these conditions, Jung identified correspondences indicative of synchronicity. Even if Jung’s statistical approach to synchronicity revealed itself inconclusive, he nevertheless decided to publish his statistical research in his synchronicity essay. It is highly plausible that Jung held on to his statistical studies to underline the necessity of approaching synchronicity through experience rather than from a purely theoretical point of view. This demonstrates Jung’s continuous attitude of empiricism which can be traced back to his earlier claims in the 1930s about being an empiricist rather than a philosopher (Jung, 1973/1992, pp. 195, 227). Therefore, Jung conceptualizes synchronicity only as an empirical claim, which does not equate to any philosophical assumption (Jung, 1952/1975, para. 995).

However, the appearance of many philosophical references (used by Jung to illustrate some conceptual prefiguration of synchronicity across the history of ideas) is striking. After two sub‐chapters mainly focused on a statistical approach to synchronicity (“Foreword and Exposition”, Jung, 1952/1975, paras. 816–871; “An Astrological Experiment”, Jung, 1952/1975, paras. 872–915), Jung dedicates an entire part of his argumentation to philosophical sources (“Forerunners of the Idea of Synchronicity”, Jung, 1952/1975, paras. 916–946). How can Jung be so reluctant about philosophy while clearly leaning on metaphysical views? Roderick Main summarizes Jung’s particular position regarding metaphysics as follows:

Jung does not deny the possible existence of a metaphysical reality, but he is wary of any claims made about such a reality that are not based on experiences amenable to psychological investigation. (Main, 2022, p. 105)

Nonetheless, Jung’s vague position regarding metaphysics contributes to the confusion surrounding the concept of synchronicity. Jung ends his essay by recognizing two potential layers of synchronicity: as “a special class of natural events” but also as “the sum of countless individual acts of creation occurring in time” (Jung, 1952/1975, para. 968). As such, the concept and its implications concerning the nature of reality continue to be very obscure. We would like to argue here that Jung thought of synchronicity as a notion to be developed and studied further as this essay aims “to open up a very obscure field” Jung, 1952/1975, para. 816). Follower and major key figure of the analytical psychology movement, Marie‐Louise von Franz proclaimed this specific task which needs to be undertaken:

I don’t know the other hopeful young men who should continue it. They must exist, but I don’t know where they are. But I think the big breakthrough had really already been done by Jung when he created the concept of synchronicity. The work which has now to be done is to work that out. It is like a lightning intuition. But now we would have to work it out in detail, explore it empirically. This could occupy a lot of people of very good mind. (von Franz, 2002, p. 144)

This task has been taken up within the context of post‐Jungian astrology. Just as the Jungian concept of archetypes has been used to rethink the principles of astrology, synchronicity is frequently conceived by contemporary astrologers as a way of explaining the nature of correlations between the movement of the stars and the corresponding human events (Howell, 1990; Grasse, 2002; Martin, 2016; Rossi & Le Grice, 2018a, pp. 1–12; Perry, 2019, pp. 58–67). However, its conception often diverged amply from Jung’s original understanding. This article aims to study the reception of synchronicity in the work of Liz Greene’s psychological astrology (1976/2011, 1978, 1983/1996, 1984, 1996a, 1996b, 1997, 2001, 2018a, 2018b) and Richard Tarnas’ archetypal astrology (2006), even though post‐Jungian astrology is not reducible to these two authors.

## Liz Greene’s Approach to Synchronicity and Qualitative Time

One of the major representatives of psychological astrology, British Jungian analyst Liz Greene, adapted Jung’s synchronicity theory to her astrological practice. Greene’s approach to synchronicity is first discussed by Maggie Hyde (1992) in the pioneering book titled *Jung and Astrology*, and recently in the unpublished theses of Zeng (2022) and by Andrikopoulos in *Jung and Twentieth Century Psychological Astrology* (2025). In this section, we will further explore Liz Greene’s astrological and Jungian writings, with an attempt to clarify her statements of synchronicity in her psychological astrology.

Liz Greene made statements about synchronicity in two ways: 1) the psychological aspect of synchronicity; 2) the physical dimension of synchronicity. In the few contexts where she talks about synchronicity and meaningful experiences, she provides explanations of the psychic aspect of the concept. However, when synchronicity is discussed in an astrological context, her arguments, though sometimes accordant, are often inconsistent with Jung’s statements about synchronicity. As we will discuss, this is one of the reasons for Greene’s predilection towards the earlier version of synchronicity that is later abandoned by Jung—synchronicity as qualitative time (Jung, 1930/1977, paras. 81–84).

In the model of qualitative time, the birth moment is believed to contain a certain quality that exists in time. Greene’s conception of synchronicity matches Jung’s initial understanding of synchronicity through qualitative time as expressed in his memorial talk for Richard Wilhelm in 1930 (Jung, 1930/1977, paras. 81–84). In this conceptualization, synchronicity as qualitative time means: “If there are any astrological diagnoses of character that are in fact correct, this is due not to the influence of the stars but to our own hypothetical time qualities” (Jung, 1930/1977, para. 82). However, Jung abandoned this idea later in his synchronicity essay in 1952, especially pondering his astrological experiment (Jung, 1952/1975, para. 986; Jung, 1958/1973, para. 1175; Rossi & Le Grice, 2018b, p. 183). He conceived from then on that the phenomenon of synchronicity is beyond the frame of time and space (Jung, 1952/1975, para. 968). He turned to the post‐Kantian hypothesis of synchronicity beyond categories including causality, time and space (Jung, 1952/1975, para. 968).

Synchronicity is discussed in detail as early as 1976 in Liz Greene’s (1976/2011) *Saturn*, an interpretive book of the astrological Saturn. As the first statement, Liz Greene (1976/2011) suggests that the awareness of new planets in astronomy as well as astrology corresponds to the emergence into the “group consciousness of the meaning of the planet” (p. 128). She then clearly asserts:

This is an example of synchronicity as Jung describes the term: the simultaneous occurrence of an inner unfoldment and an outer circumstance which have no causal connection to each other but are connected by meaning. Thus, on a mundane level, the discovery of Uranus coincided with the dawn of the electrical and industrial age as well as with two great political revolutions which resulted in the birth of a new form of government. (Greene, 1976/2011, p. 128)

In the above passage, Liz Greene argues that the collective mundane change of human society is related to the discovery of Uranus under the explanation of synchronicity. It corresponds primarily to Jung’s earlier idea that the Piscean age of Christianity in history is a synchronistic phenomenon, and subsequently to the idea of qualitative time (Jung, 1930/1977, paras. 81–84; Jung, 1959/1968, pp. 13ff). In the same book, Greene (1976/2011) also talks about Uranus as the planet related to synchronicity (p. 131). In her *Saturn* book, she discusses synchronicity from two angles: firstly, synchronicity can be revealed through the shared meaning of a human social context and a freshly discovered planet, in this case the discovery of Uranus. Secondly, synchronicity is believed to contain the same quality as the planet Uranus, as Uranus symbolizes the radical unconscious changes which happen in individuals’ lives as “chance” (Greene, 1976/2011, p. 131).

The two statements above show Greene’s attempt to recognize synchronicity as a form of psychical phenomena. Therefore, she avoids a purely metaphysical or physical explanation of astrology. Yet, in her later work, physical and metaphysical elements are confusingly mixed up. In her subsequent book, *Relating: An Astrological Guide to Living with Others on a Small Planet* (Greene, 1978), Greene developed a *synchronous model* of synchronicity related to astrological practice:

The material facts pertaining to astrology, however, such as the possibility of energy emanations from the planets which affect the energy field of the sun, are only one end of the spectrum of the archetype. The other end is symbolic, and the positions of the heavens at a particular moment in time, by reflecting the qualities of that moment, also reflect the qualities of anything born at that moment, whether it be an individual, a city, an idea, a company or a marriage. One does not cause the other; they are synchronous, and mirror each other. (Greene, 1978, p. 24)

Greene’s understanding of a synchronous correspondence between the heavenly bodies and mundane events seems inadequate to explain such stable connections as acausal or “acts of creation in time” as in Jung’s synchronicity theory (Jung, 1952/1975, para. 965). Jung claims that synchronicity is different from acausal relationships in physics (Jung, 1952/1975, para. 965). An explanation in terms of the qualities of time is an earlier version of synchronicity that is eventually abandoned by Jung as mentioned previously (Jung, 1958/1973, para. 1175; Rossi & Le Grice, 2018b, p. 183). Green evolves her conceptualization of synchronicity in astrology as a combination of physical and psychical reality with caution: “We do not know why the physical phenomenon of a wobbly and not quite spherical earth, uncertain as to its orientation in the heavens, should have any connection with the growth of human consciousness. Perhaps it is a manifestation of synchronicity on a titanic scale” (Greene, 1978, p. 266).

Liz Greene becomes more assertive about the physical connection that is already alluded to in *Relating*: “So far as the reason for this synchronicity is concerned, we are left, on the one hand, with Jung’s archetypes of the collective unconscious and, on the other, the teachings of esoteric doctrine” (Greene, 1978, p. 24). Nevertheless, her attempt is clear when she replaces Jung’s statements of synchronicity with the “teachings of esoteric doctrine”. In *Relating*, Liz Greene quoted Paracelsus’ understanding of astrology and his worldview to support her argument (Greene, 1978, p. 25). Six years later, in her book *The Outer Planets & Their Cycles: The Astrology of the Collective* (a transcript of her seminar), Greene used Hermeticism to defend her view of physical correspondence in astrology:

If I have really found some kind of pattern in Pluto’s transits through history, then the ancient Hermetic worldview ought to be on the horizon now, just getting ready to make itself felt again. Of course, I can see its tracks in Jung’s psychology, which is, in the end, built upon the same worldview after you grapple with terms like “collective unconscious” and “synchronicity” and “archetype”. Jung’s psychological vision of life, which he tried to anchor firmly in observation and empirical research, is in the end cut of the same cloth as the *Corpus Hermeticum*. … Astrology also seems to have a cyclical flowering, and not surprisingly it trots alongside Hermetic philosophy, because it’s one of the best vehicles for the vision of what is above being like what is below. (Greene, 1983/1996, pp. 24–25)

By equating the Hermetic worldview 1 and Jung’s psychology, Greene tries to make synchronicity compatible with the esoteric idea of correspondences in order to eventually support qualitative time. Even though Greene is in favour of the qualities of time in astrology, she also mentions the synchronistic significance of the consulting moment of astrological reading (Greene, 1983/1996, p. 130). Yet in the subsequent year, Greene published *The Astrology of Fate*, in which she distinctively demonstrated her understanding of physical and psychical synchronicity in astrology:

In my case, the sense of confusion and indecision as to which direction to follow constituted the abaissement which allowed the unconscious to “slip in” with its peculiar synchronistic properties. The unconscious was activated because it was the “right time”. I am convinced that such timing is inherent from the birth of the organism, just as the timing for a tomato plant to flower and produce a fruit is inherent in its nature. So, as if by chance, I encountered someone who led me into the next phase of my life, through a “meaningful coincidence” of an inner discovery and an outer event. (Greene, 1984, p. 278)

The first sentence of the passage is an affirmation of Jung’s synchronicity theory, which confirms Jung’s understanding that emotions would enhance the unconscious when one is emotionally affected (Jung, 1952/1975, paras. 856–859). Hence, the hidden archetypal and instinctive pattern becomes conscious (para. 856). However, from the second sentence on, Greene slips into the qualitative time model of astrology in which the happenings of synchronicities seem to be predictable and predetermined in one’s birth moment.

From this point onwards, Liz Greene provides a range of discussions of synchronicity on a physical dimension and a psychical dimension. The psychical dimension reappears in *The Jupiter/Saturn Conference Lectures* (Greene & Arroyo, 1984, p. 28); *The Dark of Soul: Psychopathology in the Horoscope* (Greene, 1996b, pp. 11–12); *Jung’s Studies in Astrology* (Greene, 2018a, p. 84); *The Astrological World of Jung’s* Liber Novus*: Daimons, Gods and the Planetary Journey* (Greene, 2018b, pp. 5ff). Besides that, the physical dimension can also be found hinted at in *The Astrological Neptune and the Quest for Redemption* (Greene, 1996a, p. 335); explicitly in *The Horoscope in Manifestation: Psychology and Prediction* (Greene, 1997), *Apollo’s Chariot: The Meaning of the Astrological Sun* (Greene, 2001).

Greene’s (2018b) discussion in her book on Jung’s *Liber Novus* is the most mature one. After emphasizing the importance of the forerunners of “the theory o*f correspondentia*”, she suggests that:

Referring to Goethe’s belief that synchronistic events arise from a “magical faculty of the soul” and are triggered by unexpressed emotions and passions, Jung observed: “Synchronistic (‘magical’) 2 happenings are regarded as being dependent on affects”. They are also dependent on the right astrological moment. Astrological “timing”, like magic, works because there is sympathy between Kairos—the “right moment”—and the unconscious emotional state of the individual, who, as Jung insisted, knows innately the qualitative nature of a particular moment in time, and whose unconscious psyche at that moment reflects these qualities. (Greene, 2018b, p. 117)

Her understanding and effort to put together Jung’s psychical synchronicity and physical qualities of time in astrology may exactly echo her classic statement in *The Astrology of Fate* quoted above (Greene, 1984, p. 278). The consequence of her approach is firstly to recover Jung’s cautious removal of qualitative time in astrology since the synchronicity essay (Jung, 1958/1973, para. 1175; Jung, 1976/1990, pp. 354, 464; Jung, 1977/1993, p. 462). In *The Astrology of Fate*, she seems to ignore the problems in Jung’s statistical data and turns to explain why such an experiment works. According to her, the magical power of the birth moment eventually led to the testees’ marriage which completely violates Jung’s reflection on the experiment (Jung, 1958/1973, para. 1175; Greene, 1984, p. 276). The data Jung collected is unable to provide an empirical foundation of astrology or qualitative time (Jung, 1952/1975, para. 901; Jung, 1976/1990, p. 464). Liz Greene’s astrological approach to synchronicity is an integrative approach of Jung’s different versions of synchronicity theory, although certain elements leave some theoretical confusions.

## Richard Tarnas and Synchronicity: A Cosmological Agenda

In 1991, philosopher and cultural historian Richard Tarnas published *The Passion of the Western Mind*, an account of the development of the western view of the world, going back from antiquity until contemporary thought. Later in 2006, Tarnas wrote *Cosmos and Psyche: Intimations of a New World View* focusing on the spiritual crisis of the modern Self in regard to its relation to the universe (preface, p. XV).

C. G. Jung remains one of the major influences on *Cosmos and Psyche*. Across the book, Tarnas addresses correlations between movements of the planets and human affairs in terms of archetypal dynamics. Planetary archetypes frame the development of the collective unconscious. Tarnas’ archetypal astrology/cosmology also relies on the principle of synchronicity. One might even argue that he clearly sees himself as a continuator of the “work which has now to be done” (von Franz) on synchronicity.

His chapter “Synchronicity and its Implications” (Tarnas, 2006, pp. 50–60) first summarizes Jung’s conception of synchronicity. In it, he justifies connections between planetary archetypal principles and their implications in human affairs. In the following chapter entitled “The Archetypal Cosmos” (pp. 61–70), Tarnas takes for granted his conception of synchronicity as a theoretical foundation of his archetypal understanding of the universe. The original concept of synchronicity is somehow distorted to serve the establishment of a new world view. For Tarnas, synchronicity is not only about assessing acausal connections, it is a spiritual quest that mankind has to endure to find its own meaning:

The psychological and spiritual quest of the modern self now extended beyond an exclusively subjective, intrapsychic horizon, for that quest took place within the matrix of a world that evidently possessed an intrinsic capacity for expressing and supporting meaning and purpose. Subtly and tenuously, the larger context within which the modern psyche pursued its search for wholeness had begun to shift. (Tarnas, 2006, p. 60)

He understands synchronicity as the sign of changing times, as a major shift in the history of ideas. Jung also thought of his concept as an opposition to the modern scientific paradigm of causal determinism. Indeed, he emphasized the necessity of establishing meaning as a new epistemological factor. We could not argue that Tarnas misunderstood Jung’s concept of synchronicity (his synthesis remains very faithful to the original Jungian perspective). Yet, he does not make the same use of it as Jung. The latter remained cautious about claiming that astrology should be explained by synchronicity (even though this remains the most plausible explanation). It is striking to observe that Tarnas does not mention the inconclusive outcome of Jung’s astrological experiment. However, all his observations are headed towards the assessment of a synchronistic form of astrology:

Astrology is that perspective which most directly contradicts the long‐established disenchanted and decentred cosmology that encompasses virtually all modern and post‐modern experience. It posits an intrinsically meaning‐permeated cosmos that in some sense is focused on the Earth, even on the individual human being, as the nexus of that meaning. (Tarnas, 2006, p. 63)

Nevertheless, using synchronicity to establish the legitimacy of astrology might divert the concept from its original meaning. Jung seemed to reserve the solution of synchronicity for unique and rare events. On the contrary, as soon as synchronicity becomes the frame to understand the happening of archetypal themes in the past and the future, the uniqueness of the present moment might disappear. Yet, according to Jung, synchronicity relates to a temporal coincidence between a certain psychic state and one or multiple external events which offers a parallelism with this present subjective state.

We believe the confusion comes from the fact that Jung assumed the activation of an archetype during the synchronistic connection between separated events (e.g., synchronicity happens because the golden scarab, as an archetype of rebirth, appears both in the patient’s dream and during the analytical session). However, it does not mean that archetypes are only effective because of synchronicity. It leads to a very delicate ambiguity inherent in the concept of synchronicity. This notion refers to an acausal principle which remains causal in terms of formal (archetypal) causality. For that matter, Tarnas himself is aware of this causal dimension of synchronicity:

Central to Jung’s understanding of such phenomena was his observation that the underlying meaning or formal factor that linked the synchronistic inner and outer events—the formal cause, in Aristotelian terms—was archetypal in nature. (Tarnas, 2006, p. 57)

Tarnas draws upon Jung’s lack of explanation about the causal (formal) nature of synchronicity. In this regard, his understanding of the concept is not epistemologically in contradiction with Jung’s essay. Tarnas took advantage of Jung’s imprecision on that matter. Archetypal astrology relies on synchronicity as a kind of causality (a formal one). Thus, when Tarnas thinks that planetary movements are synchronistically connected to human collective history, he seems to assume that planetary archetypes are the formal/archetypal causes of mundane events.

Tarnas’ reception of synchronicity therefore becomes incompatible with Jung’s original context. He attaches a new purpose to synchronicity in order to serve his own archetypal astrology. Synchronicity is then transformed into a metaphysical concept, an unwavering axiom shaping reality. However, this is a radical change from Jung’s original intention of establishing synchronicity as a category of experiential phenomena or acts of creation in time (that do not rely on a chronological pattern).

## Synchronicity: A Cosmological Meaning Yet to be Determined

We have almost entirely failed to establish astrology by statistical proof and even the most impressive research, such as that of Michel Gauquelin 3, has little obvious bearing on the main body of astrological practice. And if we are honest with ourselves, practical astrology is hardly as dependable as we might like it to be. (Hyde, 1992, p. 172)

Unlike Liz Greene’s inaccurate understanding of Jung’s astrological experiment, Maggie Hyde reveals the theoretical issues hidden in Jung’s synchronicity theory as well as in his astrological experiment. Hyde (1992) distinguishes two types of synchronicity: *Synchronicity I* includes an objective observer and an objectively observed synchronistic event; while *Synchronicity II* also acknowledges the observer’s subjective participation (p. 128). In the context of Jung’s astrological experiment, natal astrological data is statistically insignificant, therefore showing *Synchronicity I* cannot be found in astrology relying on the qualitative time model (Hyde, 1992, p. 29; Jung, 1952/1975, para. 901). Such a model has been contradicted by similar experiments (Jerome, 1973; Komath, 2009; Narlikar, Kunte, Dabholkar & Ghatpande, 2009). For Hyde (1992), astrologers need to embrace the nature of *Synchronicity II* by remembering Jung’s emphasis on the symbolic attitude, and should realize the importance of the act of interpretation itself (p. 208). Despite this, Hyde still suggests that Synchronicity I should be included in the practice of astrology while Synchronicity II is a type of rather occasional event compared to Synchronicity I 4 (Hyde, 1992, p. 174).

Richard Tarnas mentions the insufficiency of statistics to understand astrology: “statistical studies have added relatively little to the astrological understanding, and they appear to be methodologically inadequate for entering into the archetypal frame of reference central to the astrological tradition” (Tarnas, 2006, p. 76). He recognizes that astrology rather relies on a qualitative appreciation of reality compared to a quantitative approach as used by statistics. However, he does not focus on *Synchronicity II* (the subjective engagement of the subject who experiences a synchronistic event). Archetypal astrology only reveals correlations between archetypal themes within such events and planetary cycles. This worldview implies the predictability of synchronistic events from the past and the future. It is not an “act of creation in time”, happening in the present moment but a systematic assumption of correspondences. Eventually, one might wonder if in *Cosmos and Psyche* the unique and rare dimension of synchronicity is still taken into consideration.

In an interview in 1952, Jung stated the importance of numinosity (a term he borrowed from Rudolf Otto) as a defining quality of synchronicity: “Recently I have put a great deal of study into synchronicity (briefly, the ‘rupture of time’), and I have established that it closely resembles numinous experiences where space, time, and causality are abolished” (Jung, 1977/1993, p. 230).

One might finally argue that archetypal astrology/cosmology (as presented in *Cosmos and Psyche*) loses sight of this numinous dimension of synchronicity. If the concept of synchronicity aims to shine light on human history and collective events as a systematic premise, the mystery might be gone. Astrological correspondences relying on regular data are not conceived anymore as strange meaningful coincidences. The abnormal becomes normal. Therefore, synchronicity is not understood just as a numinous phenomenon experienced in the present by one subject, but also builds upon the assumed archetypal qualities in time. In these circumstances, the various ways of pursuing synchronicity in astrology are a phenomenon worth reflecting on, considering the qualitative time model of astrology which does not have an empirical background. We can see that the pursuit of synchronistic phenomena in astrology is an attempt for astrologers, like Greene or Tarnas, to affirm the legitimacy of astrology in this time. Nonetheless, Robert Sacco’s (2023) recent statistical research on the connections between astrological transit and synchronicity seems to disprove the relations between these two aspects.

The astrological meaning of synchronicity might rather be recovered by focusing on the act of interpretation as an act of creation in time. Although misconception exists, it will not stop further exploration of this “black tide of mud … of occultism 5” (Jung, 1963/1989, p. 150). If astrologers embrace depth psychological ideas, it is also necessary to rethink astrology in light of unconscious experience. As Hyde (1992) noted, astrology is beginning to demand recognition of the similarities between paranormal phenomena in psychoanalysis and occultism (pp. 208–209). The numinous dimension of synchronicity was somehow already expressed through the result of Jung’s astrological experiment. While there is no statistical proof of synchronicity between natal charts and traditional aspects of marriage, Jung’s *abaissement du niveau mental* allowed the activation of an archetypal factor leading to a synchronistic experience. Such an emotional charge is, according to Jung, a numinous effect of this archetypal factor inducing synchronicity (Jung, 1952/1975, para. 841). Instead of using synchronicity as a cosmological principle in astrology, the emphasis on the numinous would rather lead us to understand meaningful coincidences as parapsychological phenomena happening during the astrological reading process. Cornelius (1994/2003) points out that parapsychological phenomena may indeed be associated with the symbolic and archetypal meanings of the astrological chart at the time and place of an event in a rare case (pp. 212–214). He highlighted that the astrological symbols encountered by the astrologer at a particular moment may be associated with parapsychological phenomena, thus constituting a meaningful coincidence for the astrologer. In one of the examples discussed by Hyde (1992), she was contemplating a client’s natal chart, specifically a square aspect (which indicates conflict) between Saturn in Scorpio (Scorpio symbolizing death and the Saturn refers to restriction) and the Sun in the tenth house (the former representing an authority figure and the latter resembling the heart) (p. 190). At that very moment, she received a phone call informing her that her boss was in critical condition due to a heart attack—an event symbolized by the Sun’s placement, and its severity mirrored by the Saturn configuration. This, as Hyde noted, constitutes a parapsychological synchronistic event within the practice of astrology that: “[t]he Synchronicity II experience is partly the astrologer’s meaningful encounter with a symbol, and partly that strange feeling that the material is also about oneself” (p. 170). This may offer a way to rethink synchronicity in astrology by observing potential connections between archetypal symbols and physical and psychological realities, although this falls far short of establishing synchronicity as an explanatory principle for astrology. Further research into the relationship between synchronicity and astrology will help us to better understand the connection and differences between the two.

## Footnotes

^1^
Liz Greene (1983/1996) understands the Hermetic worldview simply as the famous paraphrase from the Emerald Tablet “as above, so below” (p. 23).

^2^
In Jung’s synchronicity essay, he distinctively asserts that synchronicity is not regressing “to a *magical causality*” (Jung, 1952/1975, para. 915).

^3^
Michel Gauquelin (1928–1991) led several statistical studies on astrological data, notably about significant correlations between birth charts of famous individuals and their excellence in their professions.

^4^
This conclusion appears somewhat peculiar, as Hyde (1992), on the one hand, underscores that Jung failed to establish the existence of Synchronicity I, yet on the other hand, still acknowledges its existence (pp. 131, 174).

^5^
In the Freud‐Jung context, the “black tide of mud … of occultism” is referring to Jung’s interest in parapsychology and precognition, which in Freud’s view means a challenge to his sexual obscurantism (Jung, 1963/1989, pp. 150–154: McGuire, as cited in Freud & Jung, 1974, p. 216).

## References

[joap70016-bib-0001] Andrikopoulos, L. (2025). Jung and twentieth century psychological astrology: Perspectives on magic, psychology and culture. Routledge.

[joap70016-bib-0002] Cornelius, G. (1994/2003). The moment of astrology: Origins in divination. The Wessex Astrologer.

[joap70016-bib-0003] Freud, S. , & Jung, C. G. (1974). The Freud‐Jung letters: The correspondence between Sigmund Freud and C. G. Jung (W. McGuire, Ed., R. Manheim & R. F. C. Hull, Trans.). Princeton University Press.

[joap70016-bib-0004] Grasse, R. (2002). Signs of the times: Unlocking the symbolic language of world events. Hampton Roads Publishing.

[joap70016-bib-0005] Greene, L. (1976/2011). Saturn: A new look to an old devil. Weiser Books.

[joap70016-bib-0006] Greene, L. (1978). Relating: An astrological guide to living with others on a small planet. Weiser Books.

[joap70016-bib-0007] Greene, L. (1983/1996). The outer planets & their cycles: The astrology of the collective. Weiser Books.

[joap70016-bib-0008] Greene, L. (1984). The astrology of fate. Weiser Books.

[joap70016-bib-0009] Greene, L. (1996a). The astrological Neptune and the quest for redemption. Weiser Books.

[joap70016-bib-0010] Greene, L. (1996b). The dark of soul: Psychopathology in the horoscope. CPA Press.

[joap70016-bib-0011] Greene, L. (1997). The horoscope in Manifestation: Psychology and prediction. CPA Press.

[joap70016-bib-0012] Greene, L. (2001). Apollo’s chariot: The meaning of the astrological sun. CPA Press.

[joap70016-bib-0013] Greene, L. (2018a). Jung’s studies in astrology: Prophecy, magic and the qualities of time. Routledge.

[joap70016-bib-0014] Greene, L. (2018b). The astrological world of Jung’s Liber Novus: *Daimons, gods and the planetary journey*. Routledge.

[joap70016-bib-0015] Greene, L. , & Arroyo, S. (1984). The Jupiter/Saturn conference lectures. CRCS Publications.

[joap70016-bib-0016] Hyde, M. (1992). Jung and astrology. The Aquarian Press.

[joap70016-bib-0017] Howell, A. (1990). Jungian synchronicity in the astrological signs and ages: Letters from an astrologer. Theosophical Publishing House.

[joap70016-bib-0018] Jerome, L. E. (1973). Astrology and modern science: A critical analysis. Leonardo, 6, 121–130.

[joap70016-bib-0019] Jung, C. G. (1930/1977). Richard Wilhelm: In memoriam. *CW* 15.

[joap70016-bib-0020] Jung, C. G. (1952/1975). Synchronicity: An acausal connecting principle. *CW* 8.

[joap70016-bib-0021] Jung, C. G. (1958/1973). An astrological experiment. *CW* 18.

[joap70016-bib-0022] Jung, C. G. (1959/1968). Aion: Researches into the phenomenology of the self. CW 9(ii).

[joap70016-bib-0023] Jung, C. G. (1963/1989). Memories, dreams, reflections (Recorded and edited by Aniela Jaffé, Richard & Clara Winston, Trans.). Vintage Books.

[joap70016-bib-0024] Jung, C. G. (1973/1992). C. G. Jung letters volume 1: 1906–1950. ( G. Adler & A. Jaffé , Eds., R. F. C. Hull, Trans.). Routledge.

[joap70016-bib-0025] Jung, C. G. (1976/1990). C. G. Jung letters volume 2: 1951–1961. ( G. Adler & A. Jaffé , Eds., R. F. C. Hull, Trans.). Routledge.

[joap70016-bib-0026] Jung, C. G. (1977/1993). C. G. Jung Speaking: Interviews and Encounters. William McGuire & R. F. C. Hull (Eds.). Princeton University Press.

[joap70016-bib-0027] Komath, M. (2009). Testing astrology. Current Science, 96(12), 1568–1572.

[joap70016-bib-0028] Rossi, S. , & Le Grice, K. (2018a). Introduction. In S. Rossi & K. Le Grice (Eds.), Jung on astrology (pp. 1–12). Routledge.

[joap70016-bib-0029] Rossi, S. , & Le Grice, K. (Eds.). (2018b). Jung on astrology. Routledge.

[joap70016-bib-0030] Main, R. (2022). Breaking the spell of disenchantment. Chiron Publication.

[joap70016-bib-0031] Martin, C. (2016). Mapping the psyche. Volume 1: The planets & the zodiac signs. Wessex Astrologer.

[joap70016-bib-0032] Narlikar, J. V. , Kunte, S. , Dabholkar, N. , & Ghatpande, P. (2009). Statistical test of astrology. Current Science, 96(5), 641–643.

[joap70016-bib-0033] Perry, G. (2019). Free will, karma and fate: Revelation of western astrology to us. Persephone Publishing.

[joap70016-bib-0034] Sacco, R. G. (2023). Do planetary transits predict synchronicity experience? Journal of Behavioral and Brain Science, 13(3), 33–44.

[joap70016-bib-0035] Tarnas, R. (2006). Cosmos and psyche: Intimations of a new world view. Penguin Publishing Group.

[joap70016-bib-0036] von Franz, M.‐L. (2002). Wolfgang Pauli, the feminine and the perils of the modern world. Interview with Marie‐Louise von Franz by Hein Stufkens and Philip Engelen, IKON‐television, Küsnacht, November 1990. Harvest: Journal for Jungian Studies, 48(2), 142–148.

[joap70016-bib-0037] Zeng, J. (2022). Contextualizing Jung’s synchronicity theory through its relation to astrology. Unpublished master’s thesis, University of Essex, UK.

[joap70016-bib-0038] Zeng, J. (2024). Jung’s conceptualization of synchronicity theory and role of astrology. International Journal of Jungian Studies, 16(2), 175–196.

